# Applications and Biological Activity of Nanoparticles of Manganese and Manganese Oxides in In Vitro and In Vivo Models

**DOI:** 10.3390/nano11051084

**Published:** 2021-04-22

**Authors:** Zuzanna Sobańska, Joanna Roszak, Kornelia Kowalczyk, Maciej Stępnik

**Affiliations:** 1 Department of Translational Research, Nofer Institute of Occupational Medicine, 8 St Teresy St., 91-348 Łódź, Poland; joanna.roszak@imp.lodz.pl (J.R.); kornelia.kowalczyk@imp.lodz.pl (K.K.); m.stepnik@qsarlab.com (M.S.); 2 QSAR Lab Ltd., Trzy Lipy 3 St., 80-172 Gdańsk, Poland

**Keywords:** manganese oxides, nanoparticles, in vitro, in vivo, biological effects

## Abstract

The expanding applications of nanotechnology seem to be a response to many technological, environmental, and medical challenges. The unique properties of nanoparticles allow for developing new technologies and therapies. Among many investigated compounds is manganese and its oxides, which in the form of nanoparticles, could be a promising alternative for gadolinium-based contrast agents used in diagnostic imaging. Manganese, which is essential for living organisms as an enzyme cofactor, under excessive exposure—for example, due to water contamination or as an occupational hazard for welders—can lead to neurological disorders, including manganism—a condition similar to Parkinson’s disease. This review attempts to summarise the available literature data on the potential applications of manganese and manganese oxide nanoparticles and their biological activity. Some of the published studies, both in vitro and in vivo, show negative effects of exposure to manganese, mainly on the nervous system, whereas other data suggest that it is possible to develop functionalised nanoparticles with negligible toxicity and novel promising properties.

## 1. Introduction

The rapidly growing field of nanotechnology opens new scientific pathways from technology to medicine. Nanoparticles (NPs) attract increasing attention since their unique and often innovative features, much different from those of their bulk forms, make them a promising tool for new solutions. NPs might be perceived as a separate substance because even a slight change in their size or shape may evidently influence their physical and biological properties [1,2,3,4]. Such an easy shift in nanomaterial characteristics creates a clear need for thorough toxicological assessment of NPs, but at the same time, it makes data analysis very difficult due to a multitude of nanoforms used in the research.

Manganese oxides, including MnO, MnO_2_, Mn_2_O_3_, and Mn_3_O_4_, whose properties predestine them as a novel magnetic resonance imaging (MRI) contrast agent and potential theranostic tool, are an example of these promising nanomaterials. The manganese itself plays a crucial biological role in mammals as an enzyme cofactor; therefore, its levels are maintained in fragile homeostasis, where both deficiency and excess of this element may be dangerous for mammals by causing neurological disturbances [5]. Therefore, medical applications of manganese oxides at different oxidation states, although attractive from the physicochemical perspective, require a thorough toxicological analysis.

Manganese (Mn) is a transition metal, existing in oxidation states of −3 to +7; however, the most common are +2, +3, +4, +6, and +7. Due to such a variety of valence states, compounds with Mn in an oxidation state of +3 or higher easily enter redox reactions. Compounds with +3, +5, and +6 valence states can undergo a disproportionation process. Compounds with divalent and tetravalent manganese are considered the most stable.

Manganese monoxide (MnO) reacts quite easily with atmospheric oxygen, forming another oxide, i.e., Mn_3_O_4_. Effectively assimilated by plants, MnO is used as a fertilizer.

Manganese dioxide (MnO_2_) is used in a battery production process due to its electrochemical properties. Moreover, depending on the reagents present in the environment, the compound can play a role of an oxidant or (in a reaction with strong alkali) can be oxidized to Mn(V) and Mn(VI) compounds [6].

Manganese, a cofactor necessary for the activity of several enzymes, plays a crucial role in human health. An optimal Mn level is needed to maintain proper cellular redox status, urea metabolism, neurotransmitter synthesis, as well as autophagy processes [5,7]. Due to the ubiquity of this element in food and water, Mn deficiency and the resulting diseases are very rare. On the other hand, the widespread use of manganese and its oxides in the industry poses an occupational risk of excessive exposure for factory workers, miners, and welders. Elevated levels of manganese in the body have been linked with neurological and neurobehavioural disturbances, including manganism, an illness with symptoms similar to Parkinson disease (PD) [8,9].

Catalytic activity of manganese and a relatively large surface area of nanoparticles (compared to bulk form) makes manganese oxide NPs a good candidate as a soil remediation agent. Effective decontamination was reported, e.g., for arsenic, selenium, thallium, cadmium, and lead species in the soil [10,11,12,13]. MnO_2_ was proven to remediate soil and water contaminated with toxic dyes, released as industrial waste [14,15] and estradiol, an endocrine disruptor chemical harmful to human and aquatic fauna [16,17,18].

Electrochemical and oxidative properties of Mn oxides predestine them to be used as a component of nanocomposites, sensitive to a broad range of compounds. MnO_2_—based biosensors can detect, i.a., α-glucosidase, glucose, or glutathione (GSH) in human blood [19,20,21] or *Salmonella typhimurium*, a pathogenic bacterium, in food products [22].

**Figure 1:**
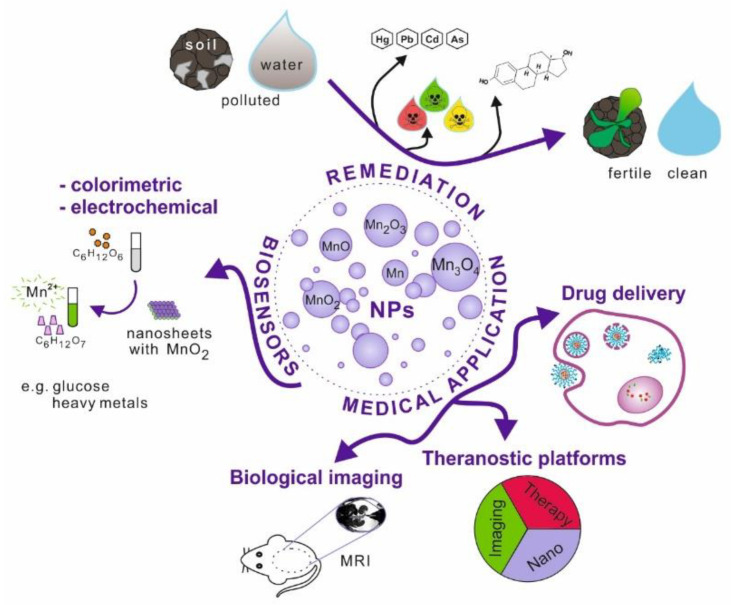
Schematic illustration of manganese and its oxides nanoparticles possible applications.

Nanoparticles, which possess unique physicochemical properties, slowly enter the field of medical applications. The emerging tools and solutions develop into new branches, e.g., nanomedicine or nanotheranostics. Nanomaterials have the potential to replace current techniques due to better biocompatibility, cell targeting ability, effective internalisation, etc. This research area is further explored in the review (Figure 1).

## 2. Medical Applications of Manganese and Manganese Oxide Nanoparticles

**Figure 2:**
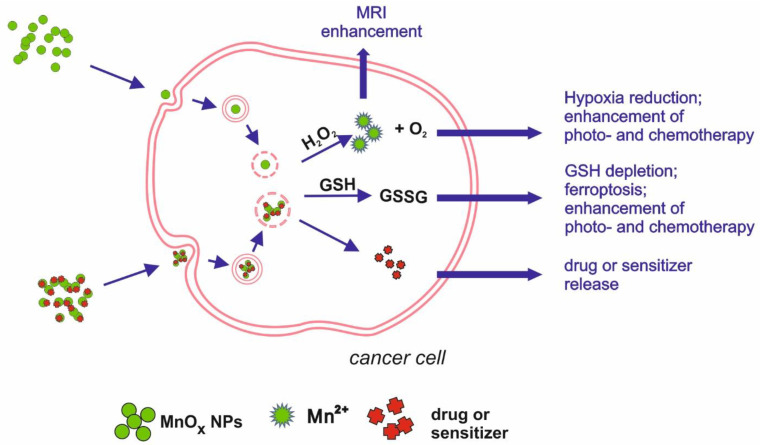
Schematic illustration of main mechanisms underlying manganese oxides medical applications.

All described applications are summarized in Table 1 and Figure 2.

### 2.1. Biological Imaging

One of the exemplary applications of Mn-oxide NPs is their potential as MRI contrasting agents (CAs). The most widely used CAs, which are based on gadolinium (Gd), are known to cause kidney fibrosis in some cases, justifying the search for a new solution. Xiao et al. [23] synthesised Mn_3_O_4_ NPs, showing high relaxivity, twice higher than that of commercially used CA, Gd-DTPA (gadolinium-diethylenetriamine penta-acetic acid). The relaxivity value specifies the ability to contrast the image by brightening specific tissues; therefore, agents with higher relaxivity can be administered at a lower dose, which increases their safety. Another nanomaterial, MnO NPs coated with polyvinylpyrrolidone (PVP), turned out to have good contrasting properties in MRI, enhancing the brightness in a dose-dependent manner [24]. Mn_3_O_4_ NPs modified with polyethylene glycol (PEG) and fluorescent dye from the cyanine group (Cy_7.5_), developed by Zhan et al. [25], allowed a dual-modality imaging based on magnetic resonance and fluorescence. The invention was intended to map and monitor sentinel lymph nodes, which is a crucial step in tumour metastasis detection, and showed effectiveness in contrasting both MRI and fluorescence images. Im et al. [26] reported the successful development of Fe_3_O_4_/MnO nanocrystals that can be used as CAs in T1- and T2-weighted images. The two modes can be used to highlight different structures, with T1 more suitable for morphological structure and T2 more suitable for pathological states. The contrasting of T1 images relies on signal enhancement (positive effect), whereas T2 contrasting agents tend to reduce the signal (negative effect). Due to their dual mode, T1 and T2 CAs have proved to be effective in differentiating the human hepatocellular carcinoma (HCC) xenograft from the surrounding liver parenchyma, which can be applied in tumour diagnosis. The mode of action involves the release of Mn^2+^ ions from the nanocrystals. The T2 signal is emitted by the entire crystal, while the ions released in a low pH environment (intracellular) are responsible for the T1 signal. As shown by the dissolution study, 53% of Mn^2+^ ions were released from the nanocrystals after 3 days of incubation in phthalate buffer solution at pH 4.6.

### 2.2. Modulating Tumour Microenvironment, Drug Delivery/Chemotherapy, Photo- and Radiotherapy

Cancer treatment is another field where Mn nanoparticles show their potential. Tang et al. [27] developed Mn-doped mesoporous silica nanoparticles to induce ferroptosis in HCC cells. Intracellular degradation of SiO_2_/Mn NPs is associated with GSH oxidation and the loss of redox balance. Moreover, the mesoporous SiO_2_/Mn NPs can be loaded with sorafenib, an anticancer drug inhibiting GSH production in the cell, released during NPs degradation. Such dually induced GSH depletion leads to ferroptosis, resulting in cell death. Other approaches to cancer treatment are photothermal therapy (PTT) and photodynamic therapy (PDT), using a high temperature or light-released ROSs to inhibit tumour growth. To induce the intracellular effect, a photosensitizer has to be administered. Nanoparticles can be loaded with such a photosensitizing agent and anticancer drugs. Moreover, they can enhance the phototherapy effects due to their physical features, e.g., acting as a photothermal agent or by depleting GSH, thus diminishing its antioxidative and protective capability [28]. Zeng et al. [29] proposed an example of such a platform, targeted at prostatic carcinoma tumours. MnO_2_ NPs were functionalized with chlorin e6 (Ce6), a photosensitizer, and polyethylene glycol-cyclic arginine-glycine-aspartic acid tripeptide (PEG-cRGD) to enhance NP biocompatibility and cell selectiveness. MnO_2_-PEG-cRGD/Ce6 NPs were effective as a photothermal and photodynamic agent, inducing in vitro hyperthermia and hypoxia after light irradiation, leading to cell death. Another complex nanoplatform was developed to target breast cancer, combining photo- and chemotherapy. Nanosized MnO_2_ combined with bovine serum albumin (BSA) was coated with IR780, a photosensitizer, and doxorubicin—an anticancer drug. MnO_2_ degradation, leading to redox imbalance, together with doxorubicin activity and photothermal therapy, were proven effective in vitro and in vivo [30]. MnO_2_ NPs were also effective in enhancing radiotherapeutic efficiency by delivering a radiosensitizer—acridine orange (AO). Under X-ray irradiation, AO promotes DNA damage, which, together with the Mn-induced release of O_2,_ produced in the presence of H_2_O_2_ (characteristic for tumour environment) and radiotherapeutically inhibited cancer cell growth, confirmed in in vitro and in vivo experiments [31]. A combined anticancer effect was achieved by developing a nanotheranostic tool, consisting of BSA base and loaded with MnO_2_ NPs and indocyanine green (ICG) [32]. ICG, delivered into tumour cells, acts as a photosensitizer, enabling photodynamic and photothermal therapy after the application of laser light. The presence of MnO_2_ leads to H_2_O_2_ disproportionation, which influences the hypoxic environment of the tumour and significantly enhances the effects of laser irradiation.

### 2.3. Theranostic Nanoplatforms

In agreement with theranostic objectives (combining diagnostics with therapy), nano-sized Mn compounds can be utilised for MRI with simultaneous drug delivery or therapeutic activity. Theranostic applications are usually complex nanoplatforms, where each compound plays a carefully planned role.

MnO nanoparticles functionalised with PEG and cyanide dye Cy_5.5_ showed not only potential as a T1-weighted MRI contrasting agent, dedicated to the myocardial infarction diagnosis, but also a good retention index in the infarcted myocardium. Therefore, loading NPs with drugs would enable a targeted therapy, making the treatment safer and reducing systemic side effects [33]. Additionally, MnO_2_ chemical properties predestine this compound to be a base for theranostic solutions. Due to the specific conditions characteristic for tumour microenvironment (TME)—low pH, high GSH level and H_2_O_2_ concentration [34,35]—MnO_2_ NPs can play a role as a carrier of chosen drug that can be released after the dissolution of NPs into Mn^2+^ [36]. Released Mn^2+^ ions can react with H_2_O_2_ present in the TME, decreasing hypoxia and changing the environment to be less favourable for tumour cells. Released oxygen can also enhance the effects of PDT. An example of such an application of MnO_2_ NPs was developed by Wang et al. [37]. Nanocomposite, produced from MnO_2_ nanosheets and verteporfin (BPD), targeted the HCC tumour cells—more specifically, tumour vessel endothelial cells (TVECs)—and released BPD, starting a coagulation cascade in the tumour vessels. The nanocomposite proved to be a good contrasting agent in multimodal imaging (MRI, fluorescence, and photoacoustic), allowing for the evaluation of tumour vessel density. Manganese oxide plays a role as a drug carrier by improving retention and tissue penetration, as well as inducing the red-ox imbalance. Bi et al. [38] designed a theranostic tool, combining chemotherapy, photodynamic therapy, and MRI imaging possibility, based on MnO_2_ nanosheets as a carrier. NPs were loaded with photosensitizer (Au25 nanoclusters) and chemotherapeutic platinum(IV) (Pt(IV)) prodrug. Apart from delivering the compounds into cancer cells, enabling multimode therapy, the reduction of manganese oxide resulted in a lower GSH concentration, which was beneficial for PDT effects. Released Mn^2+^ ions acted as T1 CA, enhancing the MRI image. Manganese oxide is an important factor of another theranostic platform, based on lyotropic liquid crystalline nanostructures (LCNs). LCNs, loaded with MnO NPs and betulinic acid (BA), were targeted at breast cancer, acting on few levels. Manganese oxide, reduced in acidic tumour environment into ions, catalyse a Fenton-like reaction, resulting in the release of ROS. In addition to oxidative stress, the breast cancer cells (4T1 and MDA-MB-231 cell lines) were exposed to BA, which enhanced the anti-tumour effect. Moreover, loaded LCNs were proven to be effective CA for MRI, combining the diagnostic and therapeutic potential [39]. Chemical features of MnO_2_ were used in nanotheranostic application, based on a tumour starvation mechanism [40]. MnO_2_ nanosheets were conjugated with glucose oxidase (GOx), enabling combined oxygenation/hyperthermia therapy as well as dual-mode MR and photoacoustic imaging. The decomposition of H_2_O_2_, present in tumour cells in high concentration, by MnO_2_ NPs releases O_2_, necessary for GOx catalytic activity. The oxidase uses up available glucose, which leads to the starvation of the cancer cells. The effect can be further enhanced by hyperthermia obtained by laser irradiation.

**Table 1:** Summary of potential medical applications of manganese oxides in nanoform.

Mn/Mn Oxide Nanoform	Modification	Application	Comments	Research Model	Reference
Mn_3_O_4_	-	MRI contrasting agent	-	Balb/c nude mice with *nasopharyngeal carcinoma* (NPC)2 xenografted tumour	Xiao et al. 2013 [23]
MnO	PVP	MRI contrasting agent	-	Human lung carcinoma cell line (SPCA-1 cells) KM mice	Hu et al. 2013 [24]
Mn_3_O_4_	PEG, Cy_7.5_	Dual modality contrasting agent (MRI + fluorescence)	-	BALB/c mice	Zhan et al. 2017 [25]
Fe_3_O_4_/MnO nanocrystals	-	MRI contrasting agent	T1 and T2 mode	BALB/c nude mice	Im et al. 2013 [26]
Mn	Doped on silica NPs	Cancer treatment + drug delivery	induce ferroptosis via GSH depletion; might be loaded with drugs, e.g., sorafenib	Human hepatocellular carcinoma cell line (HepG2)	Tang et al. 2019 [27]
MnO_2_	Ce6, PEG-cRGD	Photosensitizer delivery for PTT and PDT	-	Human prostate adenocarcinoma cell line (PC3)	Zeng et al. 2019 [29]
MnO_2_	BSA, IR780, doxorubicin	Combined photo- and chemotherapy for cancer treatment	MnO_2_ degradation leading to red-ox imbalance as additional anti-cancer mechanism	Human breast adenocarcinoma (MCF-7), Balb/c nude mice inoculated with MCF-7 tumor	Yuan et al. 2019 [30]
MnO_2_	OA	Radiosensitizer delivery	Mn-induced O_2_ release as additional anti-cancer mechanism	Human non-small cell lung cancer cell line (H1299), human head and neck squamous cell carcinoma cell line (SCC7), athymic female nude mice inoculated with H1299 cells	Liu et al. 2020 [31]
MnO_2_	BSA, ICG	Combined photothermal and photodynamic for cancer treatment	Mn-induced O_2_ release as additional anti-cancer mechanism	Nude mice inoculated with murine melanoma (B16F10) cells	Wen et al. 2020 [32]
MnO	PEG, Cy_5.5_	MRI contrasting agent + drug delivery for targeted therapy	Good retention and selectiveness	Sprague–Dawley rats with surgically developed myocardial ischemia	Zheng et al. 2018 [33]
MnO_2_	BPD	Drug delivery for targeted therapy + MRI contrasting agent	Mn-induced O_2_ release as additional anti-cancer mechanism	HepG2 orthotopic mice	Wang et al. 2020 [37]
MnO_2_	captopril–stabilized Au nanoclusters, DSP	Sensitizer (PDT) and drug delivery for targeted therapy +MRI contrasting agent	Mn ion-related depletion of GSH as mechanism supporting the effects of PDT	Mice inoculated with mouse cervical carcinoma (U14) cells	Bi et al. 2018 [38]
MnO	Loaded into LCN with BA	Chemodynamic therapy + fluorescent imaging	Mn ions catalyse Fenton-like reaction, triggering apoptosis	Balb/c mice with 4T1 (breast cancer) xenografted tumour	Urandur et al. 2020 [39]
MnO_2_	GOx	Starvation/hyperthermia therapy+ MRI and PA contrasting agent	Mn-dependent reaction releases O_2_ necessary for GOx activity	Human melanoma (A375 cells), nude mice inoculated with A375 cells	He et al. 2020 [40]

## 3. Analysis of the Biological Impact of Nano-Sized Manganese Compounds

The analysis of the data on cytotoxicity of nano-sized manganese compounds is rather difficult due to the variety of experimental setups used in the tests. Above all, the effects of nanoparticles strongly depend on their size, shape, and surface modification. Moreover, the cell lines used in in vitro studies can vary significantly in their susceptibility and response mechanisms.

Some of the studies focus on exploring whether a Mn compound in the nanoform shares the neurotoxic characteristics of the bulk form. Mn itself plays an important role as a metal cofactor of glutamine synthetase (an enzyme converting the neurotransmitter glutamate into glutamine) and superoxide dismutase (enzymatic antioxidant), and its excess results in the down-regulation of protein synthesis in neuronal cells [41]. Based on in vitro, in vivo, and human cohort studies, manganese compounds are believed, among other things, to cause neurotransmitter anomalies, elevate ROS level, and promote protein misfolding and aggregation, all the mechanisms exploited in the Parkinson’s disease model [42,43,44,45]. Therefore, the neurotoxic potential of Mn-oxide NPs requires a thorough investigation.

Another group of studies focus on the evaluation of Mn oxides NPs’ suitability for medical applications. The use of nanoparticles or nanocomposites as drug carriers or CAs requires good biocompatibility and rapid body clearance.

The fact that insoluble Mn oxides can release Mn ions when in a low pH environment is an important factor complicating Mn compound research. Therefore, the biocompatibility study should consider both aspects of Mn exposure. Additionally, the investigated compounds (various oxides in various nanoforms with surface modifications) can have different dissolution rates, which can additionally vary depending on pH [46,47,48]. This phenomenon makes analysis and comparison of the published data particularly challenging.

### 3.1. In Vitro Studies

#### 3.1.1. Studies Suggesting Cytotoxicity and Investigating Cytotoxicity Mechanisms

To compare the biological effects of manganese oxide NPs (40 nm diameter; Mn-40 nm) with Mn in the bulk form, Hussein et al. [49] treated a PC-12 cell line derived from a pheochromocytoma of the rat adrenal medulla with Mn NPs (40 nm) or manganese acetate as a source of Mn^2+^. The PC-12 cells resemble the dopaminergic neurons, having the ability to produce dopamine (DA). While the cell morphology was not altered after 24-h exposure to both manganese forms, the cell viability measured with an MTT assay was impacted by the investigated substances, with Mn NPs being the least toxic. Both Mn NPs and Mn^2+^ significantly reduced the levels of DA and its metabolites in a dose-dependent manner. Mn NPs induced oxidative stress, with an increase in ROS levels 10-fold or higher than in the control group, whereas the ROS level increased about three-fold for Mn^2+^.

Further studies [50] showed that 24-h treatment of PC-12 cells with 10 µg/mL Mn NPs (40 nm) affected the expression of genes associated with potential neurotoxicity. The expression of tyrosine hydroxylase gene, involved in dopamine synthesis, was suppressed by Mn NPs, whereas the genes associated with the DA transport system (the dopamine transporter gene (*Dat*) and vesicular monoamine transporter-2 gene (*Vmat2*)) were not impacted, suggesting that the DA depletion mechanism of action might rely on disturbances in DA production rather than transport efficiency. The Mn-NP treatment also caused the upregulation of α-synuclein gene (*Snca*) and suppression of the parkin gene (*Park2*). Both proteins play a role in PD: α-synuclein is found in Lewy bodies and is generally associated with neurodegenerative illnesses; parkin dysfunction leads to impaired ubiquitination and protein aggregation (including α-synuclein). The results suggest that the mechanisms of Mn-NP neurotoxicity might be very similar to PD pathogenesis. Another in vitro neurological model for Mn-NP investigation was N27 cell line, dopaminergic rat neurons [51]. A 9-h exposure of N27 cells to Mn NPs resulted in elevated ROS levels and a time- and dose-dependent decrease in viability. The levels of caspase-3 and protein kinase C delta type (PKCδ) proteins determined using Western Blot analysis suggests that the cell death can result from apoptosis, triggered by the proteolytic activation of PKCδ by caspase-3, intensively cleaved in response to the exposure. The cell death pathway can also shift to autophagy activation being complimentary to apoptosis. Such a scenario has been suggested by the results as showing an increase in the levels of cleaved beclin and modified LC3 protein (LC3 II)—proteins involved in autophagosome formation.

Alarifi et al. [52] exposed human neuronal cells SH-SY5Y to MnO_2_ NPs at a concentration of 10, 30, and 60 µg/mL for up to 48 h. The treatment resulted in a dose- and time-dependent viability decrease, measured with MTT and NRU assays; the cell morphology also changed. ROS levels, which were assessed with the H_2_DCFHDA probe, increased in a dose-dependent manner. The oxidative stress induction was also indicated by the elevated activity of superoxide dismutase (SOD) and catalase (CAT) and increased concentration of lipid peroxidation (malondialdehyde, MDA) products, whereas GSH concentration diminished, which can be a result of free radical release. The authors have suggested that oxidative stress can underlie the DNA damage, detected in the treated cells by comet assay. Annexin V and propidium iodide staining revealed phosphatidylserine translocation, indicating the presence of apoptotic cells. The signalling of apoptosis can be induced, e.g., by the changes in mitochondrial membrane potential (MMP) observed in the exposed cells after staining with JC-1 dye.

Chen et al. [53] focused on Mn_3_O_4_ NP cytotoxicity assessment in a PC-12 cell model. After a 24-h exposure, cell viability measured with CCK-8 and LDH release assay decreased in a dose dependent manner, and was significantly lower compared to cells exposed to a corresponding concentration of Mn^2+^. The dissolution rate for NPs was 8.8%. The NP treatment induced oxidative stress, revealed by high ROS concentration, as well as increased SOD and MDA levels and a decrease in GSH concentration. A broad panel of parameters was chosen to verify whether exposure to Mn_3_O_4_ NPs might influence the mitochondria and induce apoptosis. MMP assessment revealed differences between treated and control cells. Western Blot analysis showed an increased expression of mitochondrial calcium uniporter, a selective ion channel anchored in the mitochondrial membrane. Moreover, the concentration of Ca^2+^ ions in the mitochondria was elevated, which could lead to ROS overproduction and, ultimately, apoptosis. The apoptosis-related protein level, measured with Western Blot, was different in the exposed cells compared to the control group—the pro-apoptotic Bax protein level was elevated, whereas the level of anti-apoptotic Bcl-2 protein was suppressed. The levels of uncleaved caspase 3 and 9 also decreased, suggesting their activation and apoptosis induction, which was confirmed by staining the cells with Annexin-V and propidium iodide. Flow cytometry analysis revealed a higher percentage of apoptotic cells in the treated groups. The DA synthesis in the PC-12 cells was impacted by the treatment, which was confirmed by the measurements of the levels of DA and DA-associated proteins. The DA level was reduced significantly by the concentration of 20 µg/mL, which corresponded with a decreased expression of DOPA decarboxylase, engaged in DA production.

Frick et al. [54] investigated the influence of Mn_3_O_4_ NPs on rat type II alveolar epithelial cells (CCL-149) and compared it to the effects of water-soluble manganese sulphate. ROS production was significantly higher for NPs-treated cells as compared to Mn salt and control; the same observation concerned the level of intracellular oxidized glutathione (GSSG). However, the extracellular GSSG concentration rose for both forms of Mn, suggesting that intracellular GSSG may in part be released from the cells to keep the redox balance. TUNEL analysis showed an increased percentage of apoptotic cells at comparable concentrations of NPs and Mn salt. The results suggest that although Mn in different forms (NP or ionic) influences the glutathione homeostasis in a different way, both pathways can lead to apoptotic cell death.

Another study, aiming to compare the cytotoxic effects of MnO_2_ NPs on two epithelial cell lines derived from different tissues, was performed by Alhadlaq et al. [55]. Human breast cancer cells (MCF-7) and human fibrosarcoma cells (HT1080) were exposed to different concentrations (5–200 μg/mL) of MnO_2_ NPs for 24 h. A decrease in viability was detected using MTT, NRU, and LDH release assays in both cell lines; however, the cytotoxic effect was stronger for the HT1080 cell line (significant difference for concentrations 25 μg/mL and higher). Additionally, oxidative stress induction was higher in HT1080 cells, as revealed by ROS production, intracellular H_2_O_2_ level, and lipid peroxidation level analysis. MnO_2_ NPs treatment also influenced the antioxidative capacity of cells, which resulted in GSH depletion and decreased SOD and CAT levels. An analysis of the expression of genes associated with apoptosis revealed an upregulation of pro-apoptotic genes p53 and bax, whereas the anti-apoptotic gene bcl-2 was suppressed by the treatment. To assess the cellular uptake of NPs, Inductively Coupled Plasma Mass Spectrometry (ICP-MS) was used, revealing a higher uptake of NPs into HT1080. The authors suggested that while the cytotoxicity mechanisms for both cell lines are similar, the level of NP internalisation might underlie the difference in sensitivity between HT1080 and MCF-7 cells.

Nanozymatic capabilities of different manganese oxides regarding the redox balance were investigated by Jiang et al. [56]. MnO_2_, Mn_2_O_3_, and Mn_3_O_4_ NPs were assessed in cell-free tests for their oxidase, superoxide dismutase, and catalase mimicking activity. All compounds showed both pro-and anti-oxidative characteristics; however, their levels of activity varied. For example, Mn_3_O_4_ was proven to be the most active as GSH oxidant, while Mn_2_O_3_ was most effective in the reaction of ascorbate oxidation. The NPs were effectively internalised by human colorectal adenocarcinoma Caco-2 cells after 24 h of exposure and caused a dose-dependent decrease in cell viability. Interestingly, the cells loaded with NPs at low concentrations were less susceptible to H_2_O_2_ treatment, suggesting ROS-scavenging activity, clearly visible for MnO_2_. The protective effect diminished with growing concentrations of NPs, probably offset by the oxidase-like properties of NPs and suggesting a fragile balance between the pro- and antioxidative features of manganese oxide NPs.

#### 3.1.2. Studies Showing No Cytotoxicity of the Nanoparticles of Mn Oxides

All above data suggest that various nanosized manganese oxides can be considered as cytotoxic. Oxidative stress induction, leading to apoptosis, probably through various signalling pathways, appears to be the main mechanism underlying their toxicity. However, alongside the data pointing out the deleterious influence of Mn compounds, some studies indicate that nano-sized manganese oxides are biocompatible and safe.

Tootoonchi et al. [57] investigated the antioxidant capabilities of different manganese oxide NPs. MnO_2_ NPs were internalised by cells already after a 3.5-h exposure, but did not decrease the viability of murine insulinoma cells (bTC3) after 24 h, except for hausmannite-structured Mn_3_O_4_ in high concentrations. Moreover, MnO_2_ NPs showed a protective influence in cells treated with H_2_O_2_ for 6 h. The antioxidant abilities were stronger with increasing concentrations of NPs, up to 50 μg/mL, and then decreased, suggesting some depletion of the redox system.

Another study, analysing the usefulness of Mn_3_O_4_ NPs as an MRI contrasting agent, assessed their cytotoxicity towards three cell lines: human embryonic kidney 293 cells (HEK 293), normal nasopharyngeal epithelium cells (NP69), and human nasopharyngeal carcinoma (CNE-2). Transmission electron microscopy confirmed NPs uptake, but cell viability, measured with an MTT assay, was not impacted after 24 or 48 h of exposure. Staining with Annexin V and propidium iodide did not reveal an increased percentage of dead or apoptotic cells after the treatment, as compared to the control [23]. No cytotoxic effect was observed in another study, investigating Mn_3_O_4_ NPs functionalised with PEG and Cy_7.5_ (fluorescent dye from the cyanine group) in order to obtain better contrasting properties for MRI and fluorescent imaging. A 24-h treatment with NPs had no significant effects on the viability of human prostate cancer PC-3 cells or adenocarcinomic human alveolar basal epithelial cells A549 [25]. Another type of functional NPs, developed for enhancing MRI signal and possible drug delivery, were based on MnO conjugated with PEG-Cy_5.5_. Their cytotoxicity was tested using isolated neonatal rat ventricular myocytes (NRVMs), cardiac fibroblasts (CFs), and embryonic cardiomyocyte cells H9C2. After 48 h of incubation, the MTT assay revealed no significant differences between treated and control cells, confirming good biocompatibility of the nanoparticles [33]. MnO NPs functionalised with PVP, designed as an MRI contrasting agent, were investigated for their uptake and cytotoxicity. After a 24-h treatment of human lung carcinoma cells (SPCA-1), no differences in viability between treated and control cells were observed [24]. Cytotoxicity study, performed by Bi et al. [38] as a part of new theranostic tool development, showed no cytotoxic effect of MnO_2_ nanosheets on L929 fibroblast cells. The cells were treated for 24 h with the concentrations up to 200 µg/mL and their viability, assessed with an MTT assay, was at the level of 90–100%. Another theranostic nanocomplex, LCN loaded with MnO NPs and betulinic acid, showed no cytotoxic effect on healthy cells (human embryonic kidney cell line HEK 293) after 48 h of exposure to the concentrations equivalent to 5 and 10 µg Mn/mL, while diminishing the viability of breast cancer cells (4T1 and MDA-MB-231). Authors suggest that the discrepancy stems from the difference in pH—tumour cells create a more acidic environment, which promotes more drastic ROS induction and anti-cancer drug release, influencing cell viability [39]. MnO_2_ nanosheets combined with glucose oxidase (GOx), engineered as a thernoastic tool, were tested for cytotoxicity by He et al. [40] on human malignant melanoma (A375) cells. After 24 h of exposure to the range of 0–1 nM of NPs (MnO_2_ or MnO_2_-GOx), an MTT assay showed a mild toxic effect for higher concentrations of combined nanoparticles, whereas MnO_2_ nanosheets alone had no toxic effect.

### 3.2. Studies on In Vivo Toxicity

Toxicological evaluations primarily aim to assure the biosafety of medical applications or to estimate occupational hazards. Although in vitro studies suggest some mechanisms of toxicity, they show only a fragment of the complicated network of metabolic and genetic pathways in the living organism. Therefore, the next logical step after in vitro assessment is in vivo evaluation, better resembling the complexity of the human body. The variety of models (choice of species, length of treatment, and administration route) allow investigating different exposure scenarios. However, a thorough analysis of the available literature data provides quite contradicting results.

Oszlánczi et al. [58] administered MnO_2_ NPs intratracheally to Wistar rats on a daily basis for 3, 6, and 9 weeks. After the exposure, the behaviour of the rats was observed to assess changes in animal ambulation, rearing, and mobility. The differences were most noticeable after 9 weeks, with increased immobility and lower rearing. Electrophysiological brain activity was also influenced by the treatment, which was linked by the authors to possible dopamine and ATP depletion and mitochondrial dysfunction of neurons. The behavioural changes were correlated with significantly elevated Mn level in the brain tissue, pinpointing the fact that Mn was able to cross the blood-brain barrier. There are few theories on the mechanism of Mn translocation; one of them assumes the dissolution of insoluble particles into Mn ions, which can easily migrate. The dissolution occurs mostly after endocytosis, in macrophage phagolysosomes, where acidic pH (varying for different species) promotes the process [59]. The mechanism is called “Trojan-horse”, when insoluble NPs play the role of a carrier for ions, enabling them to enter the cell, which would be otherwise impossible for a charged particle [60]. Migration through the olfactory nerve to the olfactory bulb, as reported by Elder et al. [61], is another most likely route that enables NPs to reach the brain tissues during inhalation exposure. The authors underlined that manganese oxide nanoparticles (30 nm) used in their experiment had a dissolution rate of 1.5% at neutral pH; therefore, migration of whole NPs seems to be a major pathway of entrance to the nervous system.

In another study, investigating the influence of MnO_2_ NPs on the central nervous system, a suspension of NPs was administered intraperitoneally to Wistar rats, daily for 15 days. The treatment significantly decreased the mobility of the animals in a forced swimming test, as well as sucrose consumption—suggesting anhedonia. Depression-like behaviour was accompanied by reduced catecholamine levels in the treated group. Biochemical analysis of hippocampal homogenates revealed elevated levels of oxidative stress, manifested by increased ROS production and lipid peroxidation. These results correspond to the histopathological images of the brain tissue, showing necrotic and apoptotic foci [62]. MnO_2_, administered orally to Wistar rats, caused differences in blood biochemical parameters (LDH, alanine transaminase, and aspartate transaminase) compared to the control animals, alongside the presence of inflammation and necrotic sites in the histopathological liver, spleen, kidney, and brain images. Moreover, comet assay, micronucleus test, and chromosomal aberration assay revealed genotoxic potential of the investigated NPs. Increased levels of Mn in spleen, liver, brain, lung, and kidney tissues suggests that the manganese from the NPs can cross the gastrointestinal barrier and migrate to distant organs; however, the uptake mechanism was not investigated. Mn clearance occurred mostly through faeces. However, the researchers pointed out that the dosage in the study (30, 300, and 1000 mg/kg bw) was relatively high in order to observe clear effects [63]. The exposure of rats to Mn_3_O_4_ NPs via intraperitoneal injection (3 times a week for 6 weeks) affected many biological parameters, such as ALT, AST, and serum levels of urea, lymphocytes, granulocytes, and monocytes counts, as well as urine levels of uric acid and creatinine [64]. Histopathological examination revealed pathological changes in the liver as well as in the striatum and hippocampus tissue. An in vitro experiment showed that the biological environment (blood serum) caused significant dissolution of NPs within 24 h, with the remaining mass of NPs making up 21.2% of the initial mass, suggesting equally effective ion release in a living organism.

On the other hand, Mn_3_O_4_ NPs coated with PEG (10 ± 2.3 nm), designed as an MRI contrasting agent, showed good biocompatibility after intravenous injection in mice (20 mg/kg). Two weeks after the exposure, no visible histopathological changes were observed in the lungs, heart, spleen, liver, or kidneys as compared to the control group. Biochemical markers for liver and kidney functions (alkaline phosphatase, total bilirubin, serum creatinine, and serum urea) showed no changes. ALT and AST levels slightly decreased, but remained within the physiological range [65].

MnO_2_ nanosheets (2 nm thickness), modified with soybean phospholipid, showed good biocompatibility in Kunming mice [66]. Thirty days after an intravenous administration of NP suspension (5, 10, and 20 mg/kg), neither inflammation nor fibrosis were observed in the kidneys, heart, lung, liver, or spleen. No differences in body weight were noted, suggesting the lack of deleterious influence of the NPs. An in vitro experiment, where NPs were incubated in pH 5 or in 5 mM GSH solution (simulation of tumour microenvironment acidic and reducing conditions), showed that more than 50% of Mn ions were released from the NP structure within 10 min.

Another study focused on MnO NPs (60 nm), functionalised with PEG-Cy_5.5_ in order to enhance MRI contrast and act as a drug carrier. NPs were injected intravenously to C57BL/6J mice at the dose of 35 mg/kg bw. After 28 days, no behavioural changes in eating habits, exploring activity, etc., were noticed. Histopathological evaluation of heart, liver, spleen, kidney, lung, and brain tissue did not reveal any changes [33]. Biocompatibility study of theranostic nanoplatform, developed by Urandur et al. [39], based liquid crystalline nanoparticles loaded with MnO and betulinic acid, showed no negative effect of the nanocomplex on healthy tissues. BALB/c mice xenografted with 4T1 (breast cancer) tumours were injected intravenously with MnO, MnO + BA, and LCN loaded with MnO and BA nanoparticles. After 21 days, the animals were sacrificed and the tissues were collected for histopathological analyses. Haematoxylin and eosin staining did not reveal any pathological changes in liver, heart, lung, spleen, or kidney tissue. Histopathological analysis was also used to assess the safety of another theranostic nanocomplex, combining MnO_2_ nanosheets with glucose oxidase. Mice bearing A375 cells-derived tumour were treated with 5 mg/kg of NPs intratumorally. Tissue evaluation showed no pathological changes in healthy tissues (heart, liver, spleen, lung, kidney) after 30 days, as well as no discrepancies in body weight between treated and control groups, which suggests good biocompatibility of investigated NPs.

All data on the biological impact of manganese oxides described above are summarised in Table 2.

## 4. Conclusions

The analysis of the available data on toxicity of nano-sized Mn compounds, including both in vitro and in vivo experiments, may lead to divergent conclusions.

On the one hand, some studies show evident cyto- and genotoxicity of the investigated manganese oxide NPs, especially towards neuronal cells and the central nervous system. The main mechanism of toxicity seems to involve an induction of oxidative stress, leading to DNA damage and apoptosis. In vivo exposure was shown to cause dopamine depletion, disturbances in gene expression, and enzymatic activity.

On the other hand, other studies show good biocompatibility of nanoparticles as well as their ROS-scavenging potential, which can play a protective role at the cellular level. It is noteworthy that the studies showing low toxicity of manganese oxide NPs usually apply to chemically modified manganese oxide NPs, e.g., PEGylated or coated with PVP NPs. Considering the evident advantages of various applications of Mn compounds, whether environmental or medical, their safety should not be neglected. One of the potential solutions may be limiting the dissolution of manganese oxide NPs. Careful adjustments of size and surface characteristics of Mn oxide NPs would allow for developing nanomaterials which would ensure desired features and good biocompatibility.

## Figures and Tables

**Table 2:** Summary of in vitro and in vivo studies performed on different nanoforms of manganese and manganese oxides.

**In Vitro Studies**							
**Mn Nanoform** **(Size; Shape by TEM; Surface Modification)**	**Cell System**	**Concentration Tested; Stability**	**Exposure Time**	**Cellular Internalization**	**Toxicity Endpoints**	**Results**	**References**
Elemental Mn (40 nm; irregularly shaped cubes)	PC-12/rat pheochromocytoma	1–100 μg Mn/mL stability not measured	24 h	Effective internalization (the CytoViva 150 URI system)	Cellular morphology, cytotoxicity (MTT), ROS level; depletion of DA, DOPAC, and HVA	Moderate cytotoxicity; induction of concentration-dependent DA, DOPAC, and HVA depletion; >10-fold increase in ROS	Hussain et al. 2006 [49]
Elemental Mn (40 nm; e irregularly shaped spheres)	PC-12/rat pheochromocytoma	10 μg Mn/mL Average diameter in water: 5030 nm; in culture medium: 2390 nm.	24 h	Not measured	Expression of genes associated with dopaminergic system and redox status	Significant dopaminergic neurotoxicity; no effect on redox status related genes, suppression of Th and Park2, up-regulation of Snca (genes involved in DA metabolism and PD pathogenesis)	Wang et al. 2009 [50]
Elemental Mn (20 nm; irregular shape)	*N27*/rat dopaminergic neural *cell line*	25–400 μg Mn/mL Stability not measured	3, 6, 9 h	Effective internalization after 6 h of incubation (TEM)	Cytotoxicity (Sytox green), mitochondrial superoxide production, H_2_O_2_ induction, autophagy	Cell viability decrease, oxidative stress induction, proapoptotic protein kinase Cδ (PKCδ) cleavage, autophagy induction	Afeseh Ngwa et al. 2011 [51]
MnO_2_ (40 nm; round shape)	*SH-SY5Y/human neuroblastoma*	10, 30, 60 μg MnO_2_/mL Average diameter 299.60 nm in water	24, 48 h	Not measured	Cell morphology, cytotoxicity (MTT, NRU), MMP, ROS level, oxidative stress (LPO, GSH, SOD, CAT level), PS translocation, chromosome condensation, caspase-3 state, genotoxicity	Cell viability decrease, ROS induction, oxidative stress markers increase, apoptosis (caspase-3 activation, PS translocation, fragmentation of chromosomes)	Alarifi et al. 2017 [52]
Mn_3_O_4_ (25 nm; spheres)	PC-12/rat pheochromocytoma	5–20 μg Mn_3_O_4_/mL Average diameter 123 nm in water and 115 nm in culture medium	24 h	Not measured	Cytotoxicity (CCK-8, LDH release), intracellular ROS level, oxidative stress (SOD, MDA, and GSH), apoptosis, cytosolic Ca^2+^ concentration, MMP, apoptosis-related proteins level, DA and DA-related proteins level	Cell viability decrease, ROS induction, oxidative stress markers increase, up-regulation of Bax and suppression of Bcl-2 expression, caspase-3 and caspase-9 cleavage, cytosolic Ca^2+^ concentration increase, DA level decrease, decreased expression of DOPA decarboxylase	Chen et al. 2020 [53]
Mn_3_O_4_ (30 nm; spheres)	CCL-149/rat lung epithelium	5, 10, 20 μg Mn_3_O_4_/mL Average diameter 100 nm	24 h	Effective internalization (TEM, ICP-MS)	ROS level, GSH level, caspase-3 activity, apoptosis, LDH release	ROS induction, apoptosis, red-ox status disturbances,	Frick et al. 2011 [54]
MnO_2_ (20–120 nm; nanoflakes)	MCF-7/human breast cancer; HT1080/human fibrosarcoma	5–200 μg MnO_2_/mL Average diameter 350 nm in culture medium	24 h	Effective internalization (ICP-MS)	Cytotoxicity (MTT, NRU, LDH release), ROS level, oxidative stress markers (GSH, TBARS, SOD, CAT), apoptosis, MMP, cell cycle	Cell viability decrease and oxidative stress induction (more significant for HT1080), apoptosis, cell cycle disturbances, up-regulation of pro-apoptotic genes, and down-regulation of anti-apoptotic genes	Alhadlaq et al. 2018 [55]
MnO_2_, Mn_2_O_3_, and Mn_3_O_4_ (50 nm; spherical shape)	Caco-2/human colorectal adenocarcinoma	25 μM NP/mL Average diameter MnO_2_—232.1 nm Mn_2_O_3—_435.7 nm, Mn_3_O_4_—273 nm in water Stability not measured	24 h	Effective internalization (TEM)	Cytotoxicity (Alamar blue)	Cell viability decrease; incubation wit NPs protected from H_2_O_2_ cytotoxicity (effect decreased with growing concentrations of NPs), ROS-scavenging activity highest for MnO_2_	Jiang et al. (2020) [56]
MnO_2_, Mn_3_O_4_ (different structures)	bTC3/Murine insulinoma	3.126–200 μg NP/mL Stability not measured	48 h	Effective internalization after 3.5 h (TEM)	Cytotoxicity (bioluminescence, MTS)	Low cytotoxicity (significant for high concentrations of hausmannite Mn_3_O_4_); incubation wit NPs protected from H_2_O_2_ cytotoxicity (effect decreased from 50 μg NP/mL with growing concentrations of NPs)	Tootoonchi et al. 2017 [57]
Mn_3_O_4_ (9 nm)	Cytotoxicity: HEK 293/Human embryonic kidney cells apoptosis: NP69/Human nasopharyngeal epithelial cells CNE-2/human nasopharyngeal carcinoma cells	10, 100, 150 Mn_3_O_4_ Stability not measured	24, 48 h	Effective internalization (TEM)	Cytotoxicity (MTT), apoptosis	Cell viability unaltered, no apoptosis	Xiao et al. 2013 [23]
Mn_3_O_4_ (10 nm; spherical shape; coated with PEG and Cy_7.5_)	PC-3 (human prostate adenocarcinoma), A549 (human lung carcinoma), HepG2 (human hepatocellular carcinoma)	200–1000 μg NP/mL Stability not measured	24 h	Effective internalization (confocal microscopy)	Cytotoxicity (CCK-8)	Cell viability unaltered	Zhan et al. 2017 [25]
MnO (60 nm; coated with PEG and Cy_5.5_)	NRVM (neonatal rat ventricular myocytes), CF (cardiac fibroblasts), and H9c2 (rat myoblast)	0–100 μM NP Stability not measured	48 h	Not measured	Cytotoxicity (MTT)	Cell viability unaltered	Zheng et al. 2018 [33]
MnO (20 nm; coated with PEG)	SPCA-1 (human lung carcinoma)	0–100 μg NP/mL Average diameter 30 nm in PBS	24 h	Not measured	Cytotoxicity (MTT)	Cell viability unaltered	Hu et al. 2013 [24]
MnO_2_ (3 nm thick; nanosheets; conjugated with Au nanoclusters and DSP)	L929 (fibroblast cells)	0–200 μg NP/mL Stability not measured	24 h	Effective internalization (confocal microscopy)	Cytotoxicity (MTT)	Cell viability unaltered	Bi et al. 2019 [38]
MnO (7.3 nm;loaded into LCN wit BA)	Cytotoxicity: HEK 293 (human embryonic kidney cells) Internalization: MDA-MB-231, 4T1 (human breast cancer cells)	5, 10 μg Mn/mL	48 h	Effective internalization after 12 h (confocal microscopy, flow cytometry)	Cytotoxicity (MTT)	Cell viability unaltered	Urandur et al. 2020 [39]
MnO_2_ (2 nm thick; nanosheets; loaded with GOx)	A375 (human melanoma)	0–1 nM NP/mL Stability not measured	24 h	Not measured	Cytotoxicity (MTT, CAM + PI staining)	Cell viability unaltered	He et al. 2020 [40]
**In Vivo Studies**							
**Mn Nanoform** **(Size; Shape)**	**Animals**	**Dose Tested/Route**	**Exposure Time**	**Bio-Accumulation**	**Toxicity Endpoints**	**Results**	**References**
MnO_2_ (23 nm)	Male Wistar rats	Daily doses of 2.63 and 5.26 mg Mn/kg; intratracheal instillation	3, 6, and 9 weeks	Increased Mn level in blood and brain	Open field behaviour changes, electrophysiology, body and organ weights	Behavioural changes: increased immobility, decreased rearing, electrophysiological brain activity pattern altered, no weight gain from the 6th week on	Oszlánczi et al. [58]
MnO_2_ (30–60 nm)	Male Wistar rats	Daily doses of 50 and 100 μg MnO_2_/kg; intraperitoneal injection	15 days	Not measured	Behaviour changes, Sucrose preference, Catecholamine concentration, ROS and LPO level, histopathological analysis of tissues	Depressive-like behaviours (increased immobility, anhedonia), oxidative stress induction and catecholamine level decrease in hippocampus tissue, necrotic, and apoptotic cells in brain tissue	Sadeghi et al. 2018 [62]
MnO_2_ (42.63 nm)	Male and female Wistar rats	Daily dose of 30, 300, 1000 mg MnO_2_/kg; oral gavage	28 days	Increased Mn level in blood, liver, heart, kidneys, spleen	DNA damage (comet assay, micronucleus test, chromosomal aberration assay), blood biochemistry changes, fractionation of brain for ATPases, histopathological analysis of tissues	DNA damage, increased MN frequency and chromosome aberration for doses of 300 and 1000 mg/kg, discrepancies in brain tissue enzyme activity and blood biochemical parameters, tissue damage for the highest dose	Singh et al. 2013 [63]
Mn_3_O_4_ (~18 nm; spherical shape)	Female rats	2.5 and 1.25 mg Mn_3_O_4_/kg; 3 times a week, 18 doses in total; intraperitoneal injection	6 weeks	Increased Mn level in brain and kidneys	Behaviour changes, urine analysis, blood biochemistry and hematology changes, histopathological analysis of tissues	Discrepancies in some blood and urine parameters	Katsnelson et al. 2015 [64]
Mn_3_O_4_ (10 nm; spherical shape; coated with PEG and Cy_7.5_)	Male BALB/c mice	20 mg/kg Mn_3_O_4_; intravenously	14 days	NPs present in liver and kidneys; rapid biodegradation and clearance	Blood biochemistry, histopathological analysis of tissues	No tissue damage, no discrepancies in biochemical markers for liver and kidney functions	Zhan et al. 2018 [65]
MnO_2_ (2 nm thick nanosheets; coated with soy phospholipid)	Female Kunming mice	5, 10, 20 mg MnO_2_/kg; intravenously	30 days	Not measured	Body weight changes, histopathological analysis of tissues	No body weight discrepancies, no tissue damage	Liu et al. 2018 [66]
MnO (60 nm; coated with PEG and Cy_5.5_)	C57BL/6J mice	7 mg Mn/kg (4 and 24 h) 35 mg Mn/kg (28 h)	4 h, 24 h, 28 days	NPs present after 4, but not after 24 h; rapid clearance	Behaviour changes, histopathological analysis of tissues	No behaviour changes, no tissue damage	Zheng et al. 2018 [33]
MnO (7.3 nm; loaded into LCN wit BA)	BALB/b mice	40 mg/kg Mn; intravenously	21 days	Cleared from blood after 8 h (MnO + BA NPs) or >48 h(LCN + MnO + BA)	Histopathological analysis of tissues	No tissue damage	Urandur et al. 2020 [39]
MnO_2_ (2 nm thick; nanosheets; loaded with GOx)	Nude mice	5 mg/kg; intratumorally	30 days	Not measured	Body weight changes, histopathological analysis of tissues	No body weight discrepancies, no tissue damage	He et al. 2020 [40]

BA—betulinic acid, BPD—verteporfin, CAM—calcein AM, CAT—catalase, DA—dopamine, DOPA—levodopa, DOPAC—3,4-dihydroxyphenylacetic acid, DLS—dynamic light scattering, DSP—Pt(IV) prodrug—(*c,c,t*–Pt(NH_3_)_2_Cl_2_(OOCCH_2_CH_2_COOH)_2_, GOx—glucose oxidase, GSH—glutathione, HVA—homovanillic acid, ICG—indocyanine green, ICP-MS—inductively coupled plasma mass spectrometry, LCN—lyotropic liquid crystalline nanostructures, LDH—lactate dehydrogenase, LPO—lipid peroxidation, MDA—malondialdehyde, MMP—mitochondrial membrane potential, MN—micronucleus, MTT- 3-(4,5-dimethylthiazol-2-yl)-2,5-diphenyltetrazolium bromide, NPs—nanoparticles, PI—propidium iodide, PD –Parkinson’s disease, PS—phosphatidylserine, ROS—reactive oxygen species, SOD—superoxide dismutase, TBARS—thiobarbituric acid reactive substances, TEM—transmission electron microscopy, URI—ultra Resolution Imaging.

## Data Availability

Not applicable.
